# Fusion Potential of Human Osteoclasts In Vitro Reflects Age, Menopause, and In Vivo Bone Resorption Levels of Their Donors—A Possible Involvement of DC-STAMP

**DOI:** 10.3390/ijms21176368

**Published:** 2020-09-02

**Authors:** Anaïs M. J. Møller, Jean-Marie Delaissé, Jacob B. Olesen, Luisa M. Canto, Silvia R. Rogatto, Jonna S. Madsen, Kent Søe

**Affiliations:** 1 Clinical Cell Biology, Lillebaelt Hospital, University Hospital of Southern Denmark, 7100 Vejle, Denmark; Jean-Marie.Delaisse@rsyd.dk (J.-M.D.); Jacob.Bastholm.Olesen@rsyd.dk (J.B.O.); 2 Department of Regional Health Research, University of Southern Denmark, 5230 Odense M, Denmark; silvia.regina.rogatto@rsyd.dk (S.R.R.); Jonna.Skov.Madsen@rsyd.dk (J.S.M.); 3 Department of Clinical Biochemistry and Immunology, Lillebaelt Hospital, University Hospital of Southern Denmark, 7100 Vejle, Denmark; 4 Clinical Cell Biology, Department of Pathology, Odense University Hospital, 5000 Odense C, Denmark; 5 Department of Clinical Research, University of Southern Denmark, 5230 Odense M, Denmark; 6 Department of Molecular Medicine, University of Southern Denmark, 5230 Odense M, Denmark; 7 Department of Clinical Genetics, Lillebaelt Hospital, University Hospital of Southern Denmark, 7100 Vejle, Denmark; Luisa.Matos.Do.Canto.Alvim@rsyd.dk; 8 OPEN, Odense Patient data Explorative Network, Odense University Hospital, 5000 Odense C, Denmark

**Keywords:** osteoclast, multinucleation, osteoclastogenesis, cell fusion, DC-STAMP, CTX, aging, menopause, DNA methylation, epigenetics

## Abstract

It is well established that multinucleation is central for osteoclastic bone resorption. However, our knowledge on the mechanisms regulating how many nuclei an osteoclast will have is limited. The objective of this study was to investigate donor-related variations in the fusion potential of in vitro-generated osteoclasts. Therefore, CD14^+^ monocytes were isolated from 49 healthy female donors. Donor demographics were compared to the in vivo bone biomarker levels and their monocytes’ ability to differentiate into osteoclasts, showing that: (1) C-terminal telopeptide of type I collagen (CTX) and procollagen type I N-terminal propeptide (PINP) levels increase with age, (2) the number of nuclei per osteoclast in vitro increases with age, and (3) there is a positive correlation between the number of nuclei per osteoclast in vitro and CTX levels in vivo. Furthermore, the expression levels of the gene encoding dendritic cell-specific transmembrane protein (*DCSTAMP*) of osteoclasts in vitro correlated positively with the number of nuclei per osteoclast, CTX levels in vivo, and donor age. Our results furthermore suggest that these changes in gene expression may be mediated through age-related changes in DNA methylation levels. We conclude that both intrinsic factors and age-induced increase in fusion potential of osteoclasts could be contributing factors for the enhanced bone resorption in vivo, possibly caused by increased expression levels of *DCSTAMP*.

## 1. Introduction

Bone is a dynamic organ that undergoes continuous remodeling. To preserve bone mass and structure, bone formation levels must follow the bone resorption levels [1,2]; therefore, bone remodeling requires strict coordination between resorption and formation [3,4]. With aging and menopause, this balance shifts in a negative direction, favoring more bone resorption and less bone formation, and as a consequence, bone loss is enhanced, making the older population more prone to developing osteoporosis [1,2,5]. Osteoporosis is the main cause of fractures worldwide, which is strongly associated with both increased mortality and morbidity [5].

Bone turnover can be monitored using biomarkers, such as serum procollagen type I N-terminal propeptide (PINP) for bone formation and serum C-terminal telopeptide of type I collagen (CTX) for bone resorption [6]. CTX and PINP levels have overall been found to increase with age and for women, particularly following menopause [7,8,9,10,11,12,13,14,15]. Moreover, CTX levels have been shown to be significantly elevated in postmenopausal women with osteoporosis compared with nonosteoporotic postmenopausal women, while PINP is less elevated and may even be reduced in some cases [16]. We recently reported that monocytes from healthy female donors are “reprogrammed” in vivo, thereby allowing them to “remember” the age and menopause status of their donor, resulting in more aggressive bone-resorbing osteoclasts (OCs) in vitro [17]. This not only raises the possibility that elevated levels of bone resorption in vivo due to age and/or menopause are linked to hormonal changes [18,19,20,21,22], but also suggests that OC precursor “reprogramming” is contributing to the phenotype.

OCs are large, multinucleated cells responsible for the resorption of mineralized bone [23]. Their large size is acquired through fusion of mononuclear OC precursors (preOCs) derived from the hematopoietic lineage [24]. The fusion of preOCs and OCs is an essential step during their differentiation, ensuring effective bone resorption [25,26]. The differentiation of hematopoietic-lineage cells, monocytes, to mature multinucleated OCs in vivo depends on several structural elements, such as extracellular matrix, chemokines, and cytokines [27]. Important factors for osteoclastogenesis are, for example, macrophage colony-stimulating factor (M-CSF) [28,29], receptor activator of nuclear factor kappa-B ligand (RANKL) [29,30,31], and dendritic cell-specific transmembrane protein (DC-STAMP) [25,32,33,34,35]. DC-STAMP is a membrane-bound receptor primarily found on the plasma membrane of mononucleated preOCs [32,33]. Although a ligand for DC-STAMP remains unidentified, DC-STAMP is considered a key factor for OC fusion [25,32,33,34,35]. OC fusion does not occur at random, but fusion partners are carefully selected based on heterogeneity between them [32,36,37,38]. An example of such a heterogeneous expression pattern was identified for DC-STAMP [32]. DC-STAMP-deficient mouse bone marrow-derived preOCs demonstrated a complete abrogation of OC fusion [25]. However, DC-STAMP-deficient preOCs (DC-STAMP^lo^) were able to fuse with wild-type preOCs (DC-STAMP^hi^) [32]. These studies show that DC-STAMP is central for obtaining multinucleated OCs, but not whether DC-STAMP is also a regulator of the final number of nuclei in OCs.

There are significant variations in the onset and progression of age-induced bone loss among individuals [39,40]. The reason for this variation is, without a doubt, multifactorial. However, a plausible explanation could be that there are intrinsic differences between individuals, which, independently of structural elements, extracellular matrix, and intercellular communication [27], determine OCs’ fusion potential in vivo, and thus the final degree of nuclearity. The number of nuclei per OC is highly variable in physiological situations in vivo and in vitro. In vivo, up to 20 nuclei have been estimated, with high variability among species [41]. In pathological situations such as Paget’s disease, each OC has been shown to contain up to more than 100 nuclei [42]. Although human OCs in vitro can contain many nuclei in given experimental conditions, the vast majority of active OCs have 10 nuclei or less [38,43,44]. Several studies have shown that an increasing number of nuclei per OC results in an enhanced bone resorptive activity [17,44,45,46]. However, little is known about what regulates how many nuclei an OC will end up having.

In the current study, we used donor-related variations in the differentiation potential of in vitro-generated OCs to better understand the mechanism involved in this process. We hypothesized that one of the mechanisms by which bone resorption increases with age is through upregulating the gene expression of DC-STAMP and thereby stimulating the fusion potential of OCs. CD14^+^ monocytes were isolated from 49 healthy female blood donors (same cohort as used in our recent publication on bone resorption activity [17]), and their ability to differentiate into multinucleated OCs was investigated. Our study reveals that monocytes’ potential to differentiate into mature multinucleated OCs increases with age and is coupled to increased gene expression levels of DC-STAMP. Furthermore, the fusion potential in vitro was found to correlate with the level of in vivo bone resorption.

## 2. Results

Demographic information of the 49 blood donors included in this study is shown in Table 1. The same cohort was previously used in a study [17] showing that donor variations are associated with the basic resorptive activity of OCs generated in vitro. Here, we report how donor variations affect the degree of multinucleation of OCs in vitro according to the age, menopause status, and bone resorption levels in vivo.

### 2.1. Bone Biomarkers, CTX, and PINP Increase with Age

**Figure 1:**
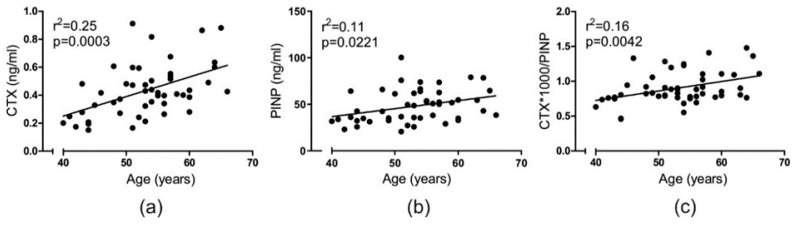
Bone resorption (CTX) and formation (PINP) increase with age. The relation between donor age and (**a**) the level of CTX (ng/mL) in vivo, (**b**) PINP (ng/mL) in vivo, and (**c**) the ratio between CTX and PINP in vivo. Statistics: correlation analyses were performed using Pearson’s correlation (r^2^). Each point represents the values for an individual donor (*n* = 49).

In a general assessment of the in vivo bone turnover characteristics of the participating blood donors, the levels of CTX (bone resorption) and PINP (bone formation) were assessed and related to their age. In this study population, the in vivo level of the bone resorption marker, CTX, was found to correlate positively (*p* = 0.0003) with increasing age (Figure 1a). Likewise, a positive correlation (*p* = 0.0221) was found between the bone formation marker, PINP, and age (Figure 1b). A ratio between bone resorption and formation levels was calculated based on the CTX and PINP levels for each donor. This ratio was found to positively correlate (*p* = 0.0042) with increasing age of the donors (Figure 1c), indicating that although both CTX and PINP individually increase with age, bone resorption levels gradually exceed the level of bone formation.

### 2.2. OCs Generated In Vitro Gain More Nuclei as Donor Age Increases

**Figure 2:**
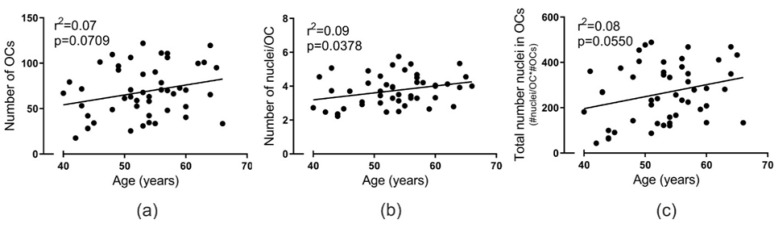
Fusion potential of OCs in vitro increases with donor age. The relation between donor age and (**a**) the mean number of OCs (per vision field), (**b**) the mean number of nuclei per OC, and (**c**) the total number of nuclei in OCs (per vision field). Statistics: correlation analyses were performed using Pearson’s correlation (r^2^). An outlier was removed in (**b**), based on Grubbs’ test for outliers (alpha = 0.01). Each point represents the results obtained from OCs in vitro generated from an individual donor (*n* = 49).

From each donor, an equal number of monocytes (5 × 10^6^ cells) were seeded in culture flasks with M-CSF and RANKL. After nine days of maturation, the number of OCs with ≥2 nuclei [17] and the mean number of nuclei per OC were manually quantified for each donor. The number of OCs and the number of nuclei per OC both reflect the precursors’ ability to differentiate into mature multinucleated OCs in vitro. The number of generated OCs did not correlate significantly (*p* = 0.0709) with the age of donors (Figure 2a); however, the number of nuclei per OC correlated positively (*p* = 0.0378) with donor age (Figure 2b). To get an arbitrary measure for the total number of nuclei found in OCs, we multiplied the mean number of nuclei per OC with the mean number of OCs (per field). The total number of nuclei in OCs reached near significance (*p* = 0.0550) when compared to the donor age (Figure 2c). Interestingly, the average number of nuclei per OC was apparently uncoupled from the number of OCs since these did not correlate (*p* = 0.1871; Figure S1).

### 2.3. Fusion Potential of OCs In Vitro Reflects In Vivo Bone Resorption Levels of Their Donor

**Figure 3:**
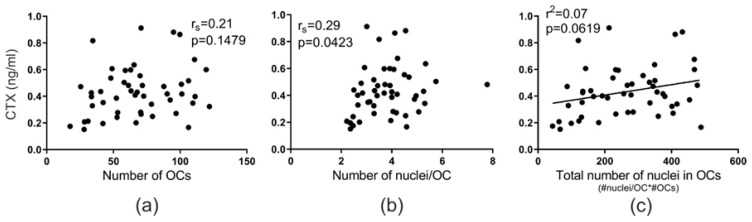
Fusion potential of OCs in vitro reflects in vivo bone resorption levels of their donor. The relation between CTX level in vivo and (**a**) the mean number of OCs (per vision field), (**b**) the mean number of nuclei per OC, and (**c**) the total number of nuclei in OCs (per vision field). Statistics: correlation analyses were performed using either Spearman’s rank correlation (r_s_) in (**a**,**b**) or Pearson’s correlation (r^2^) in (**c**). Each point represents the results obtained from OCs in vitro generated from an individual donor (*n* = 49). “#”, number; “*”, multiplied by.

CTX levels in vivo were not found to correlate with monocytes’ ability to differentiate into mature multinucleated OCs in vitro (*p* = 0.1479) (Figure 3a). However, they did correlate with the number of nuclei per OC in vitro (*p* = 0.0423) (Figure 3b). This finding suggests that individuals with high bone resorption activity in vivo also have circulating precursors in the blood that, once exposed to RANKL, fuse more readily, thereby creating OCs with a higher number of nuclei per OC. There was only a near significant correlation (*p* = 0.0619) between the level of CTX in vivo and the total number of nuclei in OCs (Figure 3c). Moreover, there was no correlation between PINP levels in vivo and the number of OCs in vitro (*p* = 0.1078), the number of nuclei per OC in vitro (*p* = 0.2758), or the total number of nuclei in OCs (*p* = 0.1173) (Figure S2a–c). Likewise, the ratio between bone resorption and formation levels was not found to correlate with the number of OCs in vitro (*p* = 0.6484), the number of nuclei per OCs in vitro (*p* = 0.1290), or the total number of nuclei in OCs (*p* = 0.2912) (Figure S2d–f).

### 2.4. Fusion Potential of OCs Determines Their Bone Resorptive Activity In Vitro

**Figure 4:**
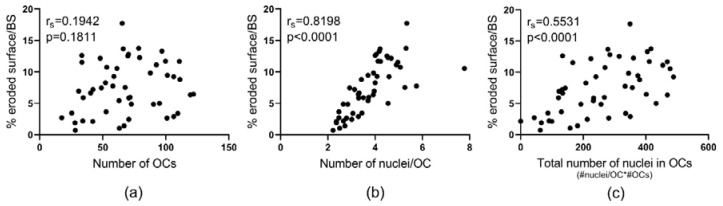
Fusion potential of OCs determines their bone resorptive activity in vitro. The relation between the percent eroded surface per bone surface in vitro and (**a**) the mean number of OCs (per vision field), (**b**) the mean number of nuclei per OC (reproduced from: Møller et al. 2020 [17] with permission of Bone Research published by Springer Nature, 2020), and (**c**) the total number of nuclei in OCs (per vision field). Of note, the numbers of OCs and nuclei were all assessed before the matured OCs were reseeded onto bone slices. Statistics: correlation analyses were performed using Spearman’s rank correlation (r_s_). Each point represents the results obtained from OCs in vitro generated from an individual donor (*n* = 49). “#”, number; “*”, multiplied by.

Monocytes’ ability to differentiate into mature multinucleated OCs in vitro did not correlate (*p* = 0.1811) with the percent eroded surface per bone surface (Figure 4a). However, a highly significant and strong correlation (*p* < 0.0001) was found between the number of nuclei per OC and the percent eroded surface per bone surface in vitro (Figure 4b). A highly significant correlation (*p* < 0.0001) was also found when comparing the total number of nuclei in OCs with the percent eroded surface per bone surface (Figure 4c). This underscores that the fusion potential of preOCs is directly linked to their bone resorptive potential.

### 2.5. DCSTAMP Expression Parallels Age, CTX Levels In Vivo, and Number of Nuclei Per OC In Vitro

**Figure 5:**
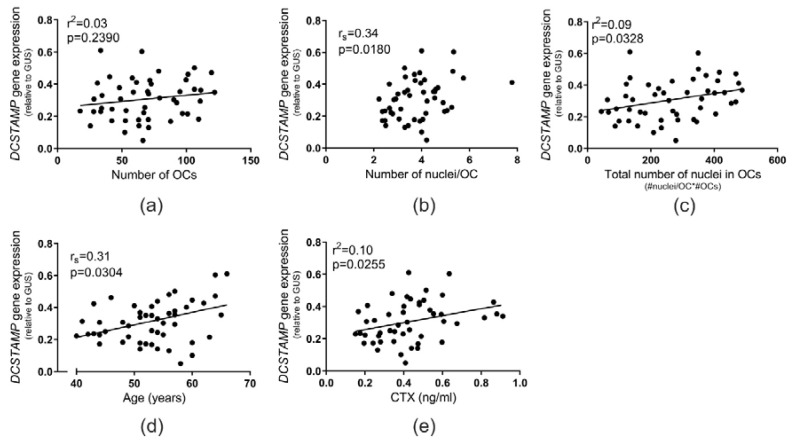
*DCSTAMP* expression levels of OCs in vitro increases with donor age, increased bone resorption activity (CTX) in vivo, and the fusion potential of in vitro-generated OCs. The relation between the gene expression of *DCSTAMP* in OCs in vitro and (**a**) the mean number of OCs (per vision field), (**b**) the mean number of nuclei per OC, (**c**) the total number of nuclei in OCs, (**d**) age (reproduced from: Møller et al. 2020 [17] with permission of Bone Research published by Springer Nature, 2020), and (**e**) the level of CTX in vivo. Statistics: correlation analyses were performed using either Spearman’s rank correlation (r_s_) in (**b**,**d**) or Pearson’s correlation (r^2^) in (**a**,**c**,**e**). Each point represents the results obtained from OCs generated from an individual donor (*n* = 49). “#”, number; “*”, multiplied by.

The gene expression level of *DCSTAMP* (*TM7SF4)*, the gene encoding DC-STAMP, was determined from OC preparations of each donor and compared to several in vivo and in vitro collected data. No correlation (*p* = 0.2390) was found between the number of OCs and *DCSTAMP* gene expression (Figure 5a). However, *DCSTAMP* gene expression showed a positive correlation (*p* = 0.0180) with the number of nuclei per OC in vitro (Figure 5b). A similar correlation was observed (*p* = 0.0328) with the total number of nuclei in OCs (Figure 5c). Remarkably, *DCSTAMP* gene expression in OCs in vitro was also found to be positively correlated with the age of the donors (*p* = 0.0304) (Figure 5d) and with their in vivo levels of CTX (*p* = 0.0255) (Figure 5e).

### 2.6. DNA Methylation Levels of Individual Sites in the DCSTAMP Gene Promoter Correlate with Age, CTX In Vivo, and the Number of OCs In Vitro

**Figure 6:**
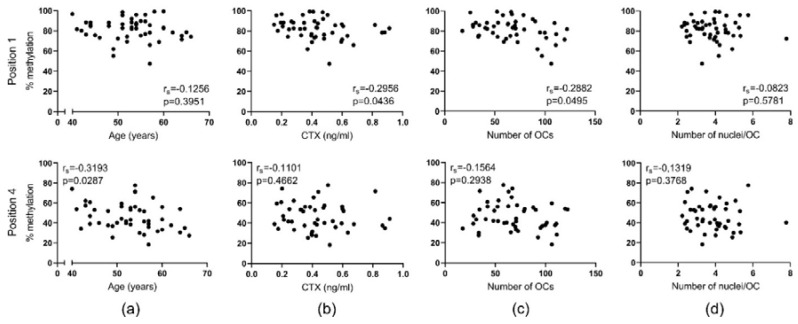
DNA methylation levels of single CpGs in the *DCSTAMP* gene decrease as donor age, bone resorption levels (CTX) in vivo, and the number of OCs in vitro increase. The relation between the level of DNA methylation of selected CpG sites in the promoter of the gene encoding DC-STAMP (*TM7SF4*) and: (**a**) age (reproduced from: Møller et al. 2020 [17] with permission of Bone Research, published by Springer Nature, 2020), (**b**) CTX levels in vivo, (**c**) the mean number of OCs (per vision field) in vitro, and (**d**) the mean number of nuclei per OC in vitro. Statistics: correlation analyses were performed using Spearman’s rank correlation (r_s_). Each point represents the results obtained from OCs generated from an individual donor (*n* = 46). The selected CpG sites are indicated as “positions”. Only relevant positions out of four analyzed were selected to be shown in this figure.

Using the same cohort, we have previously shown that: (1) hypomethylation of the CpG sites in the *DCSTAMP* gene promoter region increases its gene expression levels significantly, and (2) aging affects the DNA methylation levels of the *DCSTAMP* gene [17]. We compared the DNA methylation status of four CpGs with the donor’s age (Figure 6a). We found no correlation with the methylation status of position 1 (*p* = 0.3951), however, an inverse correlation was found with the methylation levels of position 4 (*p* = 0.0287) [17]. We therefore speculated whether these age-related changes in DNA methylation were responsible for making the preOCs inclined towards creating more highly nucleated OCs. Therefore, the DNA methylation status of the four CpGs mapped on *DCSTAMP* was evaluated and compared to the in vivo levels of CTX, the mean number of OCs in vitro, and the mean number of nuclei per OC in vitro for each donor. We found an inverse correlation for DNA methylation at position 1 (*p* = 0.0436) when compared with CTX levels in vivo (Figure 6b), while no correlation was found for position 4 (*p* = 0.4662). Moreover, the number of multinucleated OCs generated in vitro (Figure 6c) showed an inverse correlation with the CpG methylation levels at position 1 (*p* = 0.0495), whereas no correlation was observed for position 4 (*p* = 0.2938). Surprisingly, there were no significant correlations for either position 1 (*p* = 0.5781) or position 4 (*p* = 0.3768) when compared to the mean number of nuclei per OC in vitro (Figure 6d). The DNA methylation levels of CpG at positions 2 and 3 of the *DCSTAMP* gene promoter did not correlate with any of these parameters (data not shown). Our data suggest that methylation within the promoter region of the gene encoding DC-STAMP decreases with age, thereby increasing *DCSTAMP* gene expression levels. Changes in DNA methylation levels were furthermore found to be of importance for the number of generated multinucleated OCs, but not the number of nuclei per cell.

### 2.7. Menopause Status Also Has an Effect on the Fusion Potential of OCs

**Figure 7:**
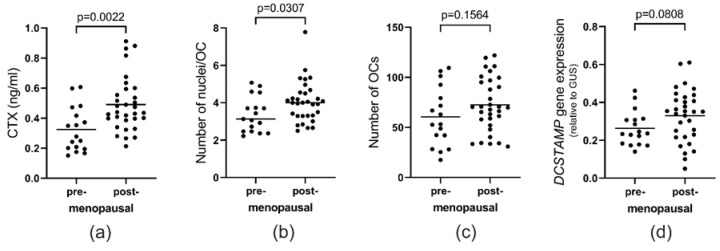
Menopause not only increases bone resorption in vivo, but also the number of nuclei per OC in vitro. Comparison between pre- and postmenopausal women regarding (**a**) the level of CTX in vivo, (**b**) the mean number of nuclei per OC in vitro, (**c**) the mean number of OCs (per vision field) in vitro, and (**d**) the gene expression level of DC-STAMP in OCs. Statistics: *t*-test in (**a**,**c**,**d**) and Mann–Whitney test in (**b**). Horizontal lines display the mean in (**a**,**c**,**d**) and the median in (**b**). Each point represents the results obtained from OCs generated from an individual donor (*n* = 49).

Considering that the ages of these women range from 40 to 66, it is difficult to differentiate an age-related effect from a menopause-related effect. Therefore, information about the donors’ menopause status was also collected. Postmenopausal women had significantly higher CTX levels (*p* = 0.0022) compared to the premenopausal women (Figure 7a). In accordance with the age effect observed in Figure 2b, postmenopausal women showed a significantly higher (*p* = 0.0307) number of nuclei per OC in vitro than premenopausal women (Figure 7b). No difference (*p* = 0.1564) in the number of OCs in vitro was found between pre- and postmenopausal women (Figure 7c). When comparing the *DCSTAMP* gene expression between pre- and postmenopausal women, a tendency was found towards a higher gene expression in postmenopausal women (Figure 7d). However, with a *p*-value of 0.0808 for the effect of menopause, the age effect (Figure 5d) appears stronger for explaining the variation in the *DCSTAMP* gene expression.

## 3. Discussion

Cell fusion is essential for the generation of mature OCs, and therefore the individual steps and mechanisms involved in OC fusion have been thoroughly investigated. It is well established that the number of nuclei is important for OCs’ bone resorptive activity, at least in vitro [17,44]. However, our knowledge is very limited concerning the mechanisms regulating how many nuclei an OC will end up having. In this study, we used the donor-related variations of OCs generated in vitro to search for mechanisms that affect this degree of nuclearity. The present data support the hypothesis that a mechanism contributing to the age-induced increase in bone resorption is through age-induced upregulation of the *DCSTAMP* gene expression and thereby the fusion potential of OCs.

We showed that CTX levels in vivo increase with age and also following menopause. PINP levels were also increased with age, though not to the same extent as CTX. These results are in accordance with a comparable Danish study population [8,9,15], as well as supported by a series of other study populations [7,10,11,12,13,14]. However, minor variations have been described among these different study populations with respect to the timing of age-induced changes in bone turnover [10,11,12,13,14,15]. Age-induced increase in bone resorption is classically interpreted as caused by hormonal changes [18,19,20,21,22]. However, the high variation in bone resorption levels in women cannot be explained by hormonal levels alone. Other factors that may be an indirect consequence of hormonal changes or independent thereof may also play a role. Recently, we described an association between the aggressiveness of OCs generated in vitro and age, menopause status, and bone formation levels [17]. Using data from the same cohort, we wanted in the present study to see if the age-increased bone resorption levels in vivo, at least in part, could be due to an increased intrinsic fusion potential of preOCs.

Our results revealed that monocytes isolated from older women have the potential to become more nucleated than monocytes isolated from younger women when stimulated with M-CSF and RANKL. These results suggest an age-induced increase in the fusion potential for monocytes isolated from healthy female donors. Monocytes from postmenopausal women were correspondingly found to have a significantly higher nucleation number than from premenopausal women. Interestingly, the bone resorptive activity of in vitro-differentiated OCs has previously been shown to correlate positively with their nucleation status [17,44,45]. Here, we further substantiate this and show strong correlations between the average number of nuclei in OCs and their resorptive activity. However, our study also shows that the nucleation status of in vitro-differentiated OCs also correlates with the bone resorption levels in vivo from the same donor. These findings suggest that the variable fusion potential of the monocyte might not only be an in vitro phenomenon, but also be a significant contributor to the age-induced increase in osteoclastic bone resorption in vivo. Besides the increased nucleation number, our results showed near significance for an age-induced increase in the number of OCs. An age-induced increase in the formation of OCs in vitro has previously been shown by Chung et al. [47] using human marrow cells from both men and women at different ages. Mouse models also showed both an age-induced [48,49] and an ovariectomy-induced increase in OC formation [50]. However, other studies found no such age-related differences in human OC formation in vitro [51,52]. These inconsistencies in results related to age and OC formation in vitro may be explained by differences in the age populations and experimental differences. Jevon et al. [51] evaluated peripheral blood mononuclear cells (PBMCs) from 22 female donors and tested the age effect by comparing the females <50 with those >50 years. However, with the age span ranging from 29 to 85 years, the chance of potential comorbidities increases, which might have added noise to the results. Furthermore, the limited number of participants and the analysis in groups may affect the outcome. Pivetta et al. [52] tested the age effect in 28 women by comparing females <50 with those >50 years, however, only six of them were over 50 years, which challenges the power of the comparison. Besides, they were testing a new technique for obtaining OC precursors from peripheral blood, which may also have affected the results [52]. In the current study: (1) we used PBMCs from 49 female donors, (2) we used an age span of 40–66 years, which centers around menopause without reaching the ages where comorbidities can be challenging to avoid, and (3) all participants were collected from the existing pool of blood donors at Lillebealt Hospital, where the participants are subjected to strict requirements in relation to diagnoses and medications, thereby decreasing the risk of “noise” from medication and comorbidities even further. Therefore, we argue that our study design and the number of participants increase the quality of our data and allow us to state that there is indeed an age-induced effect on OC precursors. Interestingly, an age-induced increase in the expression of the colony-stimulating factor-1 receptors (c-fms) and RANK receptors has also been shown [47]. This could indicate an age-dependent increase in both the total number of OC progenitors, as previously suggested [53,54], and/or the number of receptors per cell, which would be expected to increase their responsiveness to M-CSF and RANKL, as also suggested in mice [49]. Since DC-STAMP is a key factor for OC multinucleation [25,33,35] and is rapidly induced in OC precursor cells by RANKL [34], we speculated whether this age-increased fusion potential of human OCs was linked to increased *DCSTAMP* gene expression.

We found that increased gene expression of *DCSTAMP* is correlated to age, bone resorption levels in vivo (CTX), and the fusion potential of OCs in vitro. DC-STAMP is known to be heterogeneously expressed within human OC cultures in a differentiation-stage-dependent manner [33,37]. DC-STAMP was reported to be highly expressed during early OC differentiation, and to continually decrease during maturation and when the number of nuclei increases [33,37]. This may appear counterintuitive in comparison to our findings, where *DCSTAMP* gene expression increases with the average nucleation number of OCs. If DC-STAMP is primarily involved in the early fusion steps (e.g., fusion of two mononucleated preOCs), could it not be anticipated that the age-induced increase in *DCSTAMP* gene expression would result in a higher number of OCs, but with fewer nuclei? In this respect, it is relevant to note that the heterogeneous expression of *DCSTAMP* is not only an indicator for the differentiation stage of the OC, it is also essential for the selection of an appropriate fusion partner. In 2010, Mensah et al. [38] found that preOCs can be divided into two groups upon stimulation with RANKL: DC-STAMP^lo^ (larger, more nucleated OCs) and DC-STAMP^hi^ (mononuclear preOCs) cells. Interestingly, heterogeneous cultures with both DC-STAMP^lo^ and DC-STAMP^hi^ cells created larger and more nucleated OCs compared to homogeneous cultures of only DC-STAMP^lo^ cells or STAMP^hi^ cells. [32]. The authors concluded that DC-STAMP^lo^ cells displayed the “master fusogen” phenotype, whereas DC-STAMP^hi^ preOCs only acted as mononuclear donors in OC fusion [32]. In support of these findings, a live-cell imaging study on the basic fusion modalities of OCs found that more mature OCs preferentially gain nuclei by fusing with a less differentiated mononuclear preOC [38]. This principle of “acceptor/donor” cells during OC fusion has also been shown by Levaot et al., calling it “founder/follower” cells [55]. Through live-cell imaging of the fusion processes of RANKL-stimulated and nonstimulated progenitor monocytes, they showed that fusion is always initiated by a small subset of preOCs (fusion founders), and that the remaining nonstimulated precursors (fusion followers) are able to fuse with a founder cell, but not with another follower cell [55]. Altogether, these data suggest that DC-STAMP is of major importance for the multinucleation process of human OCs, and that aging affects the fusion potential of OCs by increasing the pool of DC-STAMP expressing preOCs, thereby increasing the size (number of nuclei) of the individual OC, but not necessarily the number of OCs.

In support of DC-STAMP’s importance for the fusion potential of human OCs, some interesting observations have been made in relation to Paget’s disease of bone. Paget’s disease is caused by an increase in the number and size (number of nuclei) of OCs leading to excessive bone resorption at the affected sites [56,57]. Several genome-wide association studies (GWAS) have investigated genetic loci associated with Paget’s disease of bone at the genome-wide level, and interestingly described DC-STAMP as one of them [58,59]. This is of special interest, as OC-like cells derived from patients with Paget’s disease, carrying a nonsynonymous coding variant in their *DCSTAMP* gene, display an increased number of nuclei compared to cells derived from healthy controls [60]. Combined with the fact that increased *DCSTAMP* gene expression was positively correlated with bone resorption levels in vivo (CTX), our results support the idea that an age-induced increase in the *DCSTAMP* expression leads to increased OC fusion in vitro and possibly also in vivo, thereby contributing to an enhanced bone resorptive activity of OCs both in vitro and in vivo. It is possible that these observations can be important for understanding the regulation of physiological bone resorption, but certainly also for age-induced bone loss and the development of osteoporosis. Furthermore, Coleman and colleagues in 2014 [61] published that adjuvant treatment of early breast cancer patients with zoledronic acid could reduce the risk of bone metastasis and prolong survival in postmenopausal women, thus creating a direct link to OC formation and activity in vivo. Based on this important discovery, it is interesting to speculate that our findings, with respect to regulation of osteoclastogenesis during aging, could indeed be of relevance for understanding who may be at risk for bone metastasis. However, more focused research in this regard is needed to truly evaluate the importance of our data for physiological and pathological conditions.

Since we observed that (1) in vitro-differentiated OCs “remembered” the age of the donor it was taken from as a monocyte and (2) the age-induced increase in the fusion potential appeared to be relevant for both in vitro-differentiated OCs and possibly OCs in vivo, we speculated whether the age-induced change in *DCSTAMP* gene expression could be the result of age-related changes in DNA methylation levels of the *DCSTAMP* gene. This appeared as a plausible mechanism since aging, lifestyle, and environmental influences have already been found to induce a general drift in DNA methylation, primarily resulting in hypomethylation [62,63]. Besides, both menopause [62,63] and osteoporosis [64,65,66,67,68] have been linked to a general increase in hypomethylation. In the current study, we show that the DNA methylation status of one CpG (position 1) site in the promoter of the gene encoding DC-STAMP decreased with age [17], while the DNA methylation status of another CpG site (position 4) decreased with (1) increasing numbers of in vitro-differentiated OCs and (2) increasing CTX levels in vivo. Together, these data suggest that age reprograms preOCs for an increased fusion potential by epigenetic changes in DNA methylation levels of the gene encoding DC-STAMP. However, our study is not sufficient to conclude definitively on this. Therefore, more studies are needed to clarify it further.

In summary, we found that the fusion potential of OCs generated in vitro from 49 healthy female blood donors was related to donor age, menopause status, and bone resorptive activity in vivo. We propose that an age-induced increase in the fusion potential may be a contributing factor for age-induced increase in bone resorption levels in vivo. Moreover, *DCSTAMP* gene expression levels were found to be positively correlated to all of the following parameters: CTX levels in vivo, age, and degree of nuclearity of OCs in vitro. Additionally, changes in DNA methylation levels of specific CpG sites in the promoter of the gene encoding DC-STAMP suggest that an age-induced increase in fusion potential may be mediated by epigenetic regulation. However, further studies are needed to better understand the interplay of age, epigenetics, and preOC reprogramming, and to understand the possible clinical impact of these findings.

## 4. Materials and Methods

### 4.1. Participants, Sample Collection, and Demographics

A 500 mL blood donation was collected from 50 healthy female blood donors (between 40 and 66 years of age). The blood donation was fractioned, and the buffy coat was collected. Approximately two weeks later (mean: 12.8 days, median: 14 days), a fasting blood sample was collected by standard venipuncture procedure. To obtain serum, the blood samples were allowed to clot at room temperature and were centrifuged at 2000× *g* for 10 min, and immediately stored at minus 80 °C until further use. Demographics, lifestyle information, and medical history were collected using questionnaires. The basic characteristics of the study population are summarized in Table 1. We have previously used the same study population to report on another recent study [17]. Exclusion criteria: prior bisphosphonate treatment and fractures within the last two years. One participant was excluded due to technical issues during the purification of CD14^+^ monocytes. All participants were recruited from the existing blood donor corps at Lillebaelt Hospital, Vejle, Denmark, and were therefore all considered “healthy”, as they are subjected to strict health requirements by law. Eight participants presented minor medical conditions such as hypothyroidism (*n* = 3), ulcers (*n* = 2), and asthma/allergy (*n* = 3).

### 4.2. CTX-I and PINP Measurements

As previously described, serum levels of CTX and PINP were measured using the semi-automated Cobas E602 analyzer [17]. Routine diagnostic analyses were carried out at the Department of Clinical Biochemistry and Immunology, University Hospital of Southern Denmark-Vejle (accredited by Danish Accreditation Fund (DANAK) according to the ISO 15189 standard).

### 4.3. In Vitro Generation of Human OCs

From each buffy coat, CD14^+^ monocytes were isolated and differentiated to mature OCs, as previously described [17,36]. In brief, CD14^+^ cells were purified from the pool of PBMCs by using anti-human CD14 magnetic particles (BD Biosciences, San Jose, CA, USA). Subsequently, cells were seeded at a density of 5 × 10^6^ cells in T75 culture flasks (Greiner, Frickenhauser, Germany) and differentiated into mature OCs over nine days with M-CSF and RANKL (R&D Systems, Abingdon, UK), as previously described [17,69,70]. Following the nine days of maturation, 12 systematic and evenly distributed pictures of the unstained OCs were taken using phase contrast with a ckx41microscope with an SC30 camera (Olympus Corporation, Tokyo, Japan). From these phase contrast pictures (please refer to [17] for examples of phase contrast images), the number of OCs with ≥2 nuclei [17] and the number of nuclei/OC were manually quantified in a blinded manner. Please also refer to [17] where Supplementary Information 3 shows (based on the same dataset) a very good correlation between tartrate-resistant acid phosphatase activity and the average number of nuclei/OC.

### 4.4. Bone Resorption Assays and Quantification of Bone Resorption

The matured OCs were carefully detached from T75 culture flasks by accutase treatment (PSS, Pasching, Austria) and were reseeded onto cortical bovine bone slices (Boneslices.com, Jelling, Denmark) at a density of 50,000 cells/bone slice with 25 ng/mL M-CSF and 25 ng/mL RANKL as described in [17]. The experiment was terminated after 72 h of incubation at 37 °C, 5% CO_2_, and in a humidified atmosphere. Bone resorption events were stained using toluidine blue (Sigma-Aldrich, St. Louis, MO, USA) and bone resorption was quantified by assessing the percentage of eroded surface per bone surface for all bone slices as described in [17]. Quantifications were performed in a systematic random manner using an Olympus BX50 microscope (Olympus Corporation, Tokyo, Japan) with a 100-point grid (Pyser optics, Edenbridge, UK) in the ocular. Bone slices within individual experiments were blinded by a colleague prior to quantification, meaning that the observer was fully blinded during the analysis.

### 4.5. Droplet Digital RT-PCR

Cells from each donor were lysed and RNA was isolated using the Trizol Plus RNA Purification kit (Invitrogen, Carlsbad, CA, USA), as described in [71]. cDNA was generated using the iScript cDNA synthesis kit (BioRad, Hercules, CA, USA) followed by ddPCR, which was performed as previously described [17]. In brief, the copy number concentrations were measured by ddPCR (QX100™ Droplet Digital™ PCR system, BioRad). The absolute quantification of PCR targets was analyzed using QuantaSoft™ software version 1.3.2.0 (BioRad) following the instructions of the supplier. The expression of the target genes was normalized to GUS. All TaqMan primer sets were used according to instructions by the manufacturer (Applied Biosystems, Foster City, CA, USA), GUS: Hs99999908_m1 (ViC-MGB), DC-STAMP: Hs00166156_m1 (FAM/MGB).

### 4.6. DNA Methylation Analyses Using Pyrosequencing

The DNA methylation status of four CpGs mapped in the *DCSTAMP* gene promoter was evaluated using pyrosequencing, as previously described [17]. In brief, genomic DNA was extracted using the QIAamp DNA Mini Kit (Qiagen, Valencia, CA, USA), and purity and concentration were determined using the Nanodrop 1000 spectrophotometer (Thermo Scientific, Waltham, MA, USA). A total of 500 ng DNA was bisulfite-converted using EZ DNA Methylation-Gold™ Kit (Zymo Research, Irvine, CA, USA). Subsequently, DNA was amplified using the PyroMark PCR kit (Qiagen). The resulting PCR products were checked using the D1000 ScreenTape on the Agilent TapeStation (Agilent Technologies, Glostrup, Denmark), and sequenced on the PyroMark Q24 system (Qiagen). Primer sequences used for PCR and pyrosequencing: *DCSTAMP*_Forward: TGTTTGGGGTTATGAGTGTAG, *DCSTAMP*_Reverse: [biotinylated] TTACCCTCACTCCCATACT, *DCSTAMP* _Sequencing: GGTTATGAGTGTAGAGG.

### 4.7. Statistics

Graphs and associated statistics were performed using GraphPad Prism software version 8 (GraphPad software, San Diego, CA, USA). Normal distribution was investigated using the D’Agostino and Pearson test. Correlations were identified using Spearman’s rank correlation (r_s_) or Pearson’s correlation (r^2^). Comparisons between groups were completed using a *t*-test or a Mann–Whitney test. Grubbs’ test for outliers was used to exclude one outlier in Figure 2b (alpha = 0.01). Statistical significance was defined as *p* < 0.05. Each point represents the results obtained from OCs generated from an individual donor. All figures were prepared using CorelDRAW X5 (Corel Corporation, Ottawa, ON, Canada).

### 4.8. Study Approval

This protocol was approved by The Scientific Ethical Committee for the Region of Southern Denmark with approval number S-20150059, approved on the 6 July 2015. Written informed consent was obtained from all participants. Participants were pseudo-anonymized by giving them a specific number, which they were subsequently identified by.

### 4.9. Data Availability

The datasets generated and/or analyzed during the current study are available from the corresponding author on reasonable request.

## Figures and Tables

**Table 1:** Demography of 49 female blood donors ^1^.

	Clinical Features	*n*	%
**Age**	40–44	8	16.3
	45–49	6	12.2
	50–54	15	30.6
	55–59	11	22.5
	60–66	9	18.4
**Menopause Status**	Premenopausal	17	34.7
	Postmenopausal	32	65.3
**Smoking Status**	Nonsmoker	42	85.7
	Smoker	7	14.3
**Comorbidity**	No	41	83.7
	Yes	8	16.3
	Hypothyroidism	3	
	Asthma/Allergy	3	
	Ulcers	2	
	**Median (IQR** ^2^ **) [Range]**		
**Years since Menopause**	3.5 (8.25) [0; 23]	49	
	**Mean (SD** ^3^ **) [Range]**		
**Age**	53.0 (6.7) [40; 66]	49	
**Age of Premenopausal Women**	46.4 (5.0) [40; 54]	17	
**Age of Postmenopausal Women**	56.5 (4.9) [45; 66]	32	
**Height (m)**	1.70 (0.6) [1.56; 1.84]	49	
**Weight (kg)**	73.2 (13.35) [55; 124]	49	
**BMI** ^4^	25.4 (4.0) [19.5; 37.8]	49	

^1^ Reproduced from: Møller et al. [17] with permission of Bone Research, Springer Nature, 2020, ^2^ Interquartile Range, ^3^ Standard Deviation, ^4^ Body Mass Index.

## Supplementary Materials

The following are available online at https://www.mdpi.com/1422-0067/21/17/6368/s1. Figure S1: The number of nuclei per OC is independent of the number of OCs generated in individual cultures; Figure S2: Bone formation in vivo (PINP) does not correlate with the number of OCs (per vision field) or the number of nuclei per OC in vitro.

## Abbreviations

CTX	C-terminal telopeptide of type I collagen
DC-STAMP	Dendritic cell-specific transmembrane protein
*DCSTAMP*	Gene encoding “Dendritic cell-specific transmembrane protein”
M-CSF	Macrophage colony-stimulating factor
OC	Osteoclast
PINP	Procollagen type I N-terminal propeptide
RANKL	Receptor activator of nuclear factor kappa-B ligand
