# Supplementary material for: Fusion Potential of Human Osteoclasts In Vitro Reflects Age, Menopause, and In Vivo Bone Resorption Levels of Their Donors—A Possible Involvement of DC-STAMP
Source: Int J Mol Sci. 2020 Sep 2;21(17):6368. doi: 10.3390/ijms21176368 (PMC7504560; doi:10.3390/ijms21176368)
Supplement: Supplementary file 1 [file ijms-21-06368-s001.pdf]

Supplementary Materials

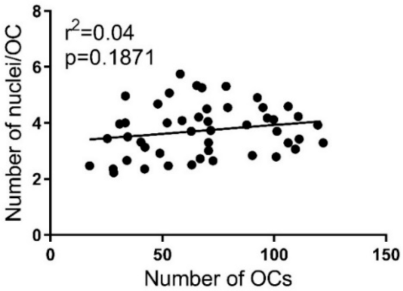

Figure S1. The number of nuclei per OC is independent of the number of OCs generated in individual cultures. The relation between the mean number of OCs (per vision field) and the mean number of nuclei per OC. Statistics: correlation analysis was performed using Pearson's correlation (r^2). Each point represents the results obtained from OCs generated from an individual donor (n = 49).

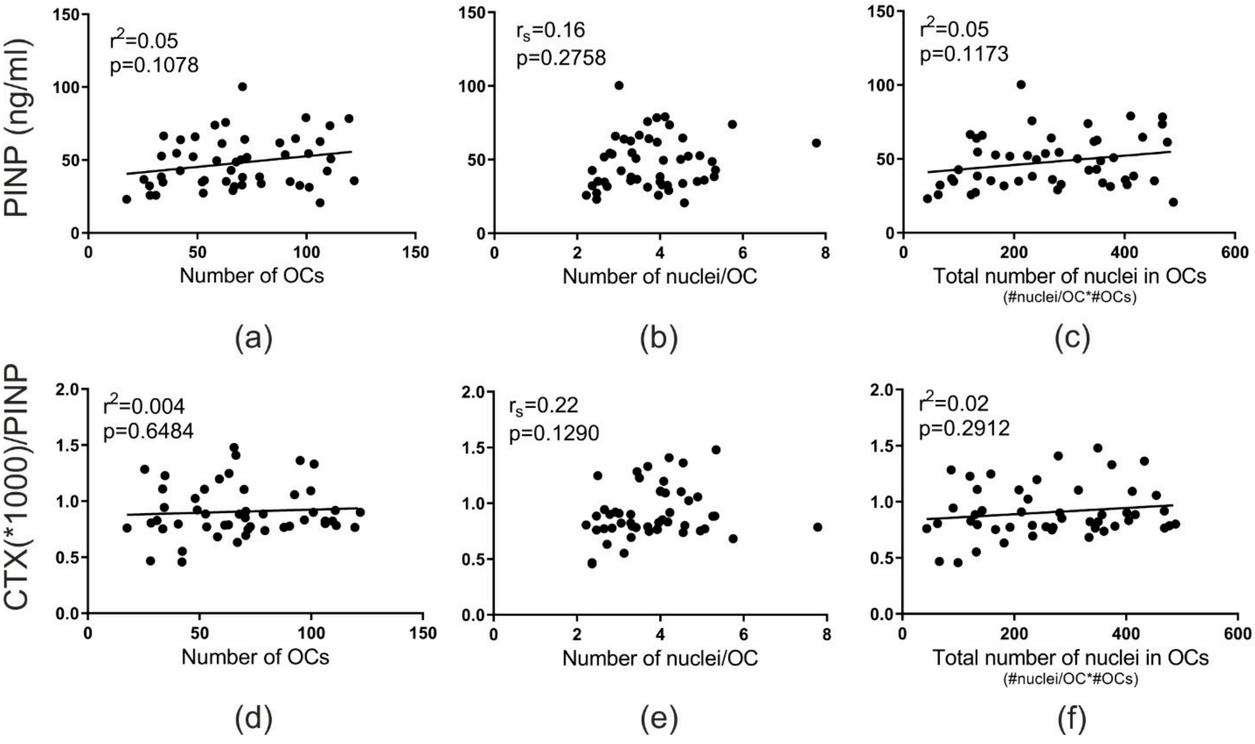

Figure S2. Bone formation in vivo (PINP) does not correlate with the number of OCs (per vision field) or the number of nuclei per OC in vitro. Comparison of the PINP levels in vivo with (**a**) the number of OCs (per vision field) in vitro, (**b**) the number of nuclei per OC in vitro, (**c**) and the total number of nuclei in OCs (per vision field) in vitro. Comparison of the ratio between CTX and PINP in vivo with (**d**) the number of OCs (per vision field) in vitro, (**e**) the number of nuclei per OC in vitro, (**f**) and the total number of nuclei in OCs (per vision field) in vitro. Statistics: correlation analyses were performed using Spearman's rank correlation (r_s) in (**b**) and (e) or Pearson's correlation (r^2) in (**a**), (**c**), (**d**), and (**f**). Each point represents the results obtained from OCs generated from an individual donor (n = 49).
